# Ulcerative cutaneous sarcoidosis without systemic involvement: An isolated presentation

**DOI:** 10.1016/j.jdcr.2026.08.008

**Published:** 2026-08-17

**Authors:** Kayla Brockmeyer, Simona A. Alomary, Ananya Udyaver, Lauren Banner, Alessio Gianni, Sweta Subhadarshani

**Affiliations:** a Department of Dermatology, Sidney Kimmel Medical College at Thomas Jefferson University, Philadelphia, Pennsylvania; b Rutgers New Jersey Medical School, Newark, New Jersey; c Department of Dermatology, Perelman School of Medicine at the University of Pennsylvania, Philadelphia, Pennsylvania

**Keywords:** cutaneous sarcoidosis, general dermatology, isolated ulcerative cutaneous sarcoidosis, medical dermatology, ulcerative cutaneous sarcoidosis

## Introduction

Sarcoidosis, a chronic granulomatous inflammatory disease of unknown etiology, typically affects the lungs and skin. Cutaneous involvement is seen in 25% to 30% of cases.^1^ Ulcerative cutaneous sarcoidosis (UCS) is a rare variant, most often manifesting in pretibial areas of females with darker skin phenotypes.^2^^,^^3^ Here, we present a patient with ulcerating nodules of the lower extremities as the initial manifestation of sarcoidosis, without preexistence of systemic sarcoidosis.

### Case presentation

A 53-year-old female with hypertension and obesity presented for evaluation and treatment of violaceous subcutaneous nodules on both lower extremities. The lesions initially began as macules that gradually became raised, ulcerated, and crusted, with periods of intermittent pain and pruritus. Prior to the onset of these lesions, the patient reported an episode of severe bilateral lower extremity edema 2 years prior, associated with intense pruritus and pain. This resolved with mechanical compression and topical corticosteroids.

Physical examination revealed scattered and grouped violaceous-to-erythematous subcutaneous nodules on the lower legs, several with overlying crust and focal ulceration, with associated edema and areas of post-inflammatory atrophic change (Fig 1). Punch biopsies of the right lower leg were obtained for histopathologic evaluation and tissue culture.

**Fig 1:**
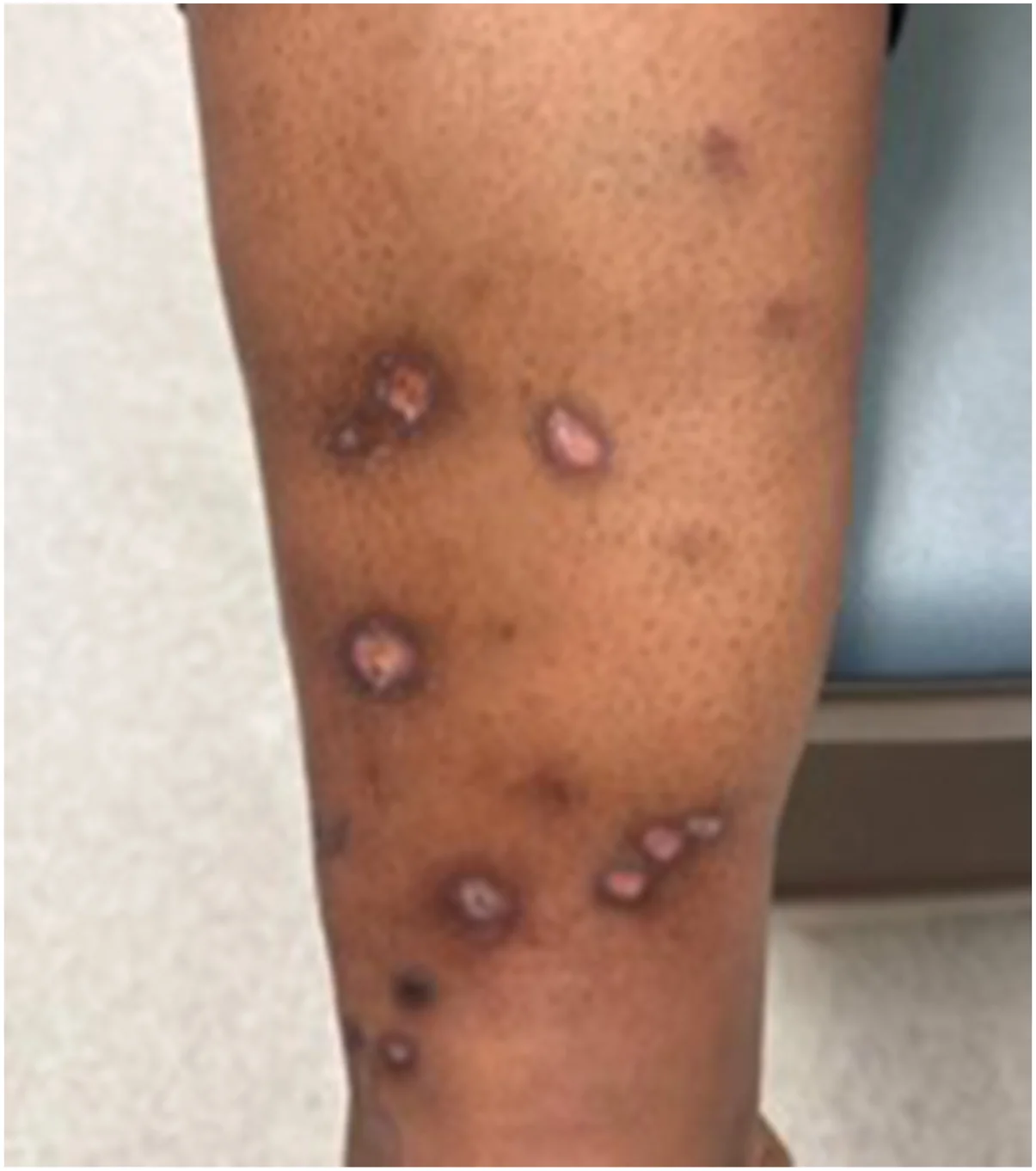
Ulcerative cutaneous sarcoidosis. Initial presentation of the right lower extremity demonstrating scattered and grouped violaceous-to-erythematous subcutaneous nodules. Some lesions contain overlying crust and focal ulceration, and there is associated edema and areas of post-inflammatory atrophic change.

Histopathologic evaluation demonstrated granulomatous inflammation (Fig 2), prompting further evaluation for sarcoidosis. Periodic Acid-Schiff, Acid-Fast Bacillus, and Fite stains failed to reveal organisms, and no foreign body was detected on polarized microscopy. Bacterial, fungal, and mycobacterial tissue cultures were negative. Laboratory workup was normal including a serum angiotensin-converting enzyme level and interferon-gamma release assay. Chest radiography was unremarkable. The patient was started on clobetasol ointment. At a follow-up visit 1 month later, she reported no new lesions yet continued to experience pain due to ulceration. She was started on a 40 mg prednisone taper and referred to pulmonology for systemic evaluation. Pulmonology obtained computed tomography chest imaging, pulmonary function testing, serum angiotensin-converting enzyme and calcitriol levels, and transthoracic echocardiography, all of which were unremarkable. The patient was also referred to ophthalmology to evaluate for ocular involvement; the results of this evaluation were pending at the time of manuscript preparation. At subsequent follow-up, her ulcerations improved following treatment with prednisone.

**Fig 2:**
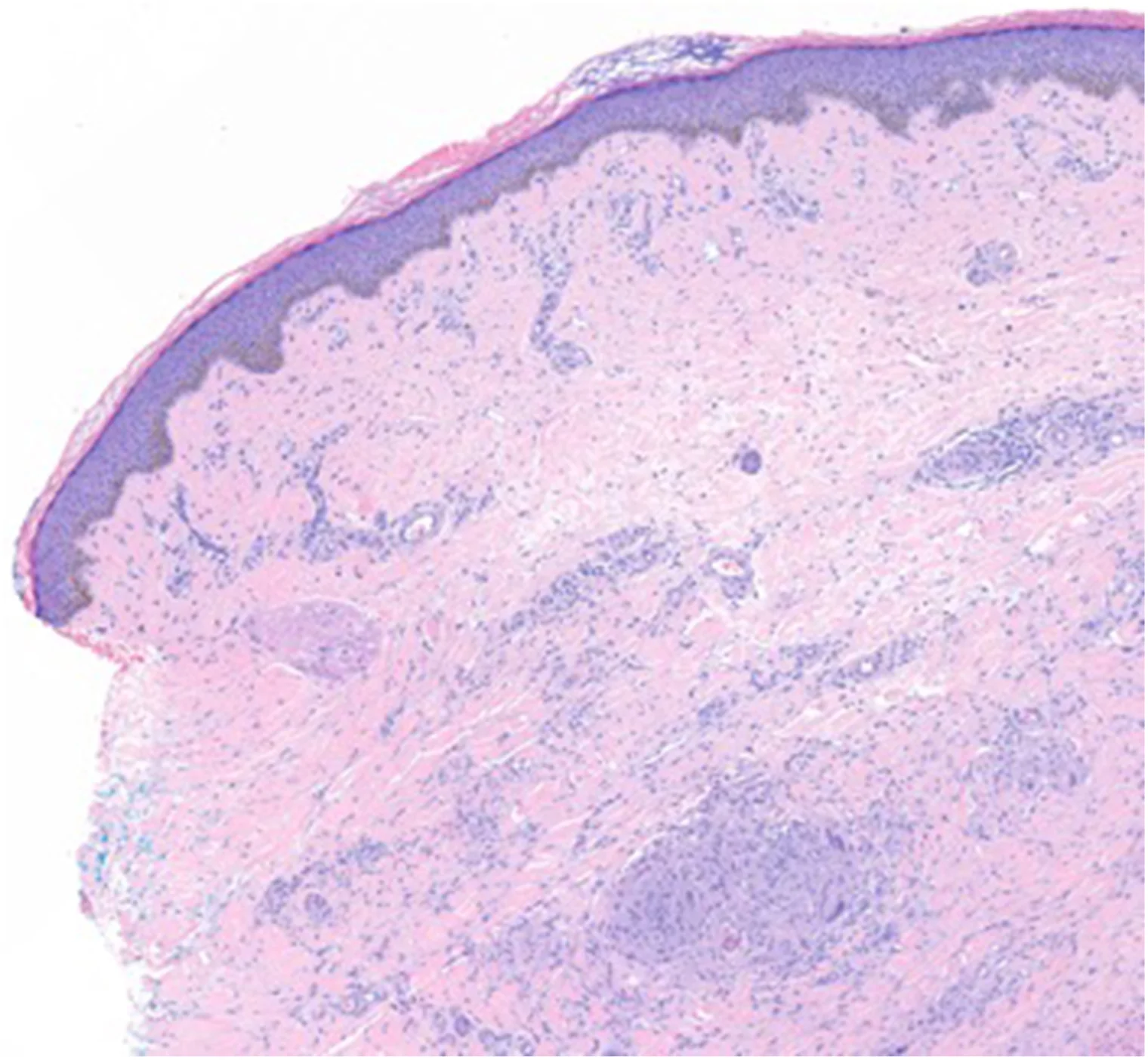
Histopathology of cutaneous sarcoidosis. Punch biopsy with histopathologic evidence of granulomatous inflammation, noncaseating epithelioid granulomas with sparse lymphocytic infiltrate, consistent with sarcoidosis. H&E, 10×.

## Discussion

Herein, we present a case of cutaneous limited ulcerative sarcoidosis, a variant rarely described in prior literature. Typically, ulcerative lesions are diagnosed after a diagnosis of systemic sarcoidosis is made.^4^ However, in this case, it is unclear whether UCS will progress to coexist with systemic sarcoidosis. Thus, continued laboratory evaluation, annual chest x-ray, and follow up with appropriate specialty services are imperative to evaluate for potential systemic transformation.

In the absence of systemic findings, ulcerative cutaneous sarcoidosis is a rare and diagnostically challenging presentation, often mimicking panniculitis, necrobiosis lipoidica, lipodermatosclerosis, vasculopathy, or atypical mycobacterial infection.^2^, ^3^, ^4^, ^5^ The most common clinical patterns of UCS are necrotic yellow plaques or violaceous nodules that eventually ulcerate, as seen in our patient.^2^, ^3^, ^4^, ^5^ Erythematous macules with ulceration, crust, and pruritus involving the trunk have also been described.^5^

Skin biopsy typically reveals non-caseating granulomas composed of epithelioid histiocytes, lacking an area of central caseating necrosis. Clinicopathologic correlation and appropriate microbiologic studies are imperative in order to exclude tuberculosis and other infectious granulomatous diseases, particularly given reports of necrotizing granulomas in UCS.^2^ Lower extremity edema is uncommon and may reflect granulomatous infiltration of lymphatics.^5^

In previously reported cases of ulcerative cutaneous sarcoidosis, trauma to atrophic plaques has been proposed as a mechanism of ulceration.^5^^,^^6^ The ulcers appear to be refractory in nature, but treatment success has been reported with individual or combination therapy with systemic corticosteroids, hydroxychloroquine, mycophenolate mofetil, methotrexate, thalidomide, and tumor necrosis factor-alpha inhibitors.^2^^,^^4^^,^^7^ In previous studies, systemic corticosteroids have been shown to be effective in around 30% of cases.^2^ Even in the absence of systemic involvement, ongoing surveillance is warranted.

With this case, we highlight the importance of recognizing variable presentations of cutaneous sarcoidosis such as UCS. This is particularly important in patients without a diagnosis of systemic sarcoidosis, as cutaneous findings may be the initial presentation. Dermatologists should perform thorough evaluation for systemic involvement in all patients with a new diagnosis of cutaneous sarcoidosis.

### Declaration of generative AI and AI-assisted technologies in the writing process

Not applicable.

## Conflicts of interest

Subhadarshani is a PI for AstraZeneca and EMD Serono.
